# Supplementary figures and images for: From free text to clusters of content in health records: an unsupervised graph partitioning approach
Source: Appl Netw Sci. 2019 Jan 24;4(1):2. doi: 10.1007/s41109-018-0109-9 (PMC6400329; doi:10.1007/s41109-018-0109-9)

## Types of accidents

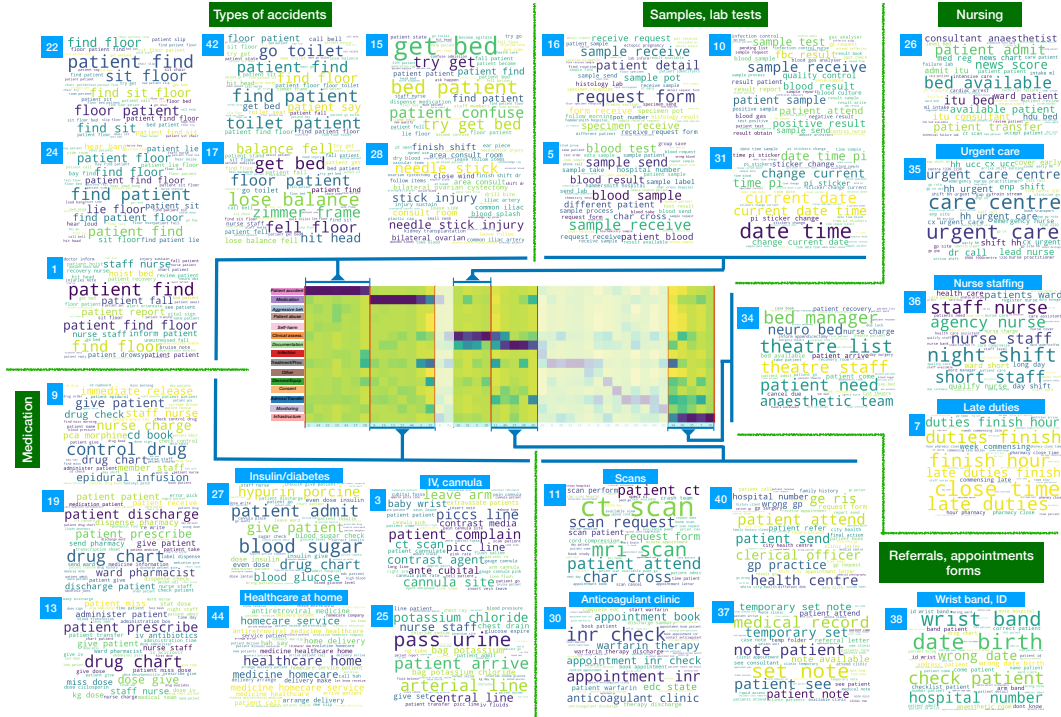

# B

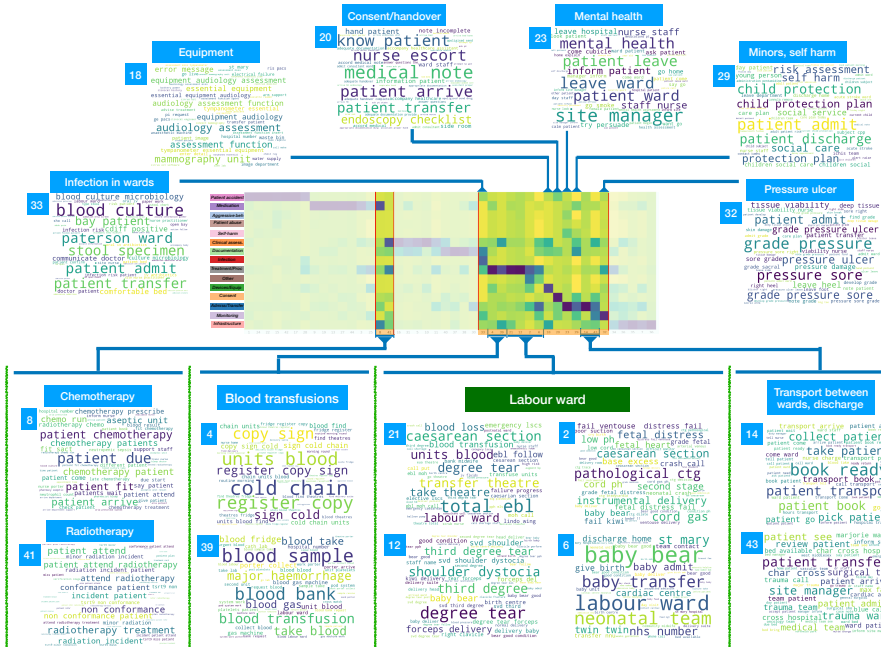

Supplement: Supplementary file 1 — Word clouds for the 44 community partition. (PDF 209 kb) [file 41109_2018_109_MOESM1_ESM.pdf]

**17 communities**

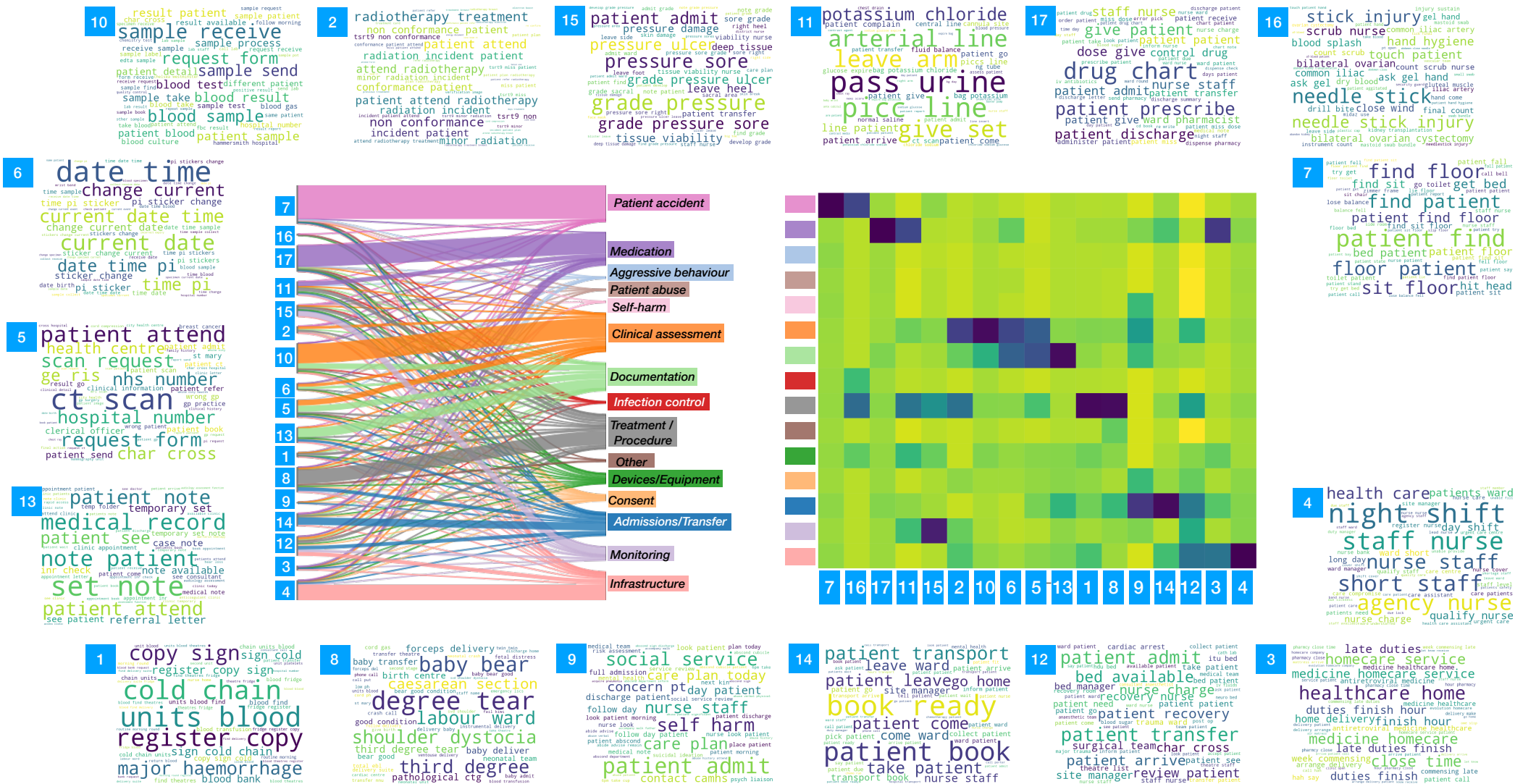

Supplement: Supplementary file 2 — Word cloud and Sankey diagram for the 17 community partition. (PDF 169 kb) [file 41109_2018_109_MOESM2_ESM.pdf]

**A**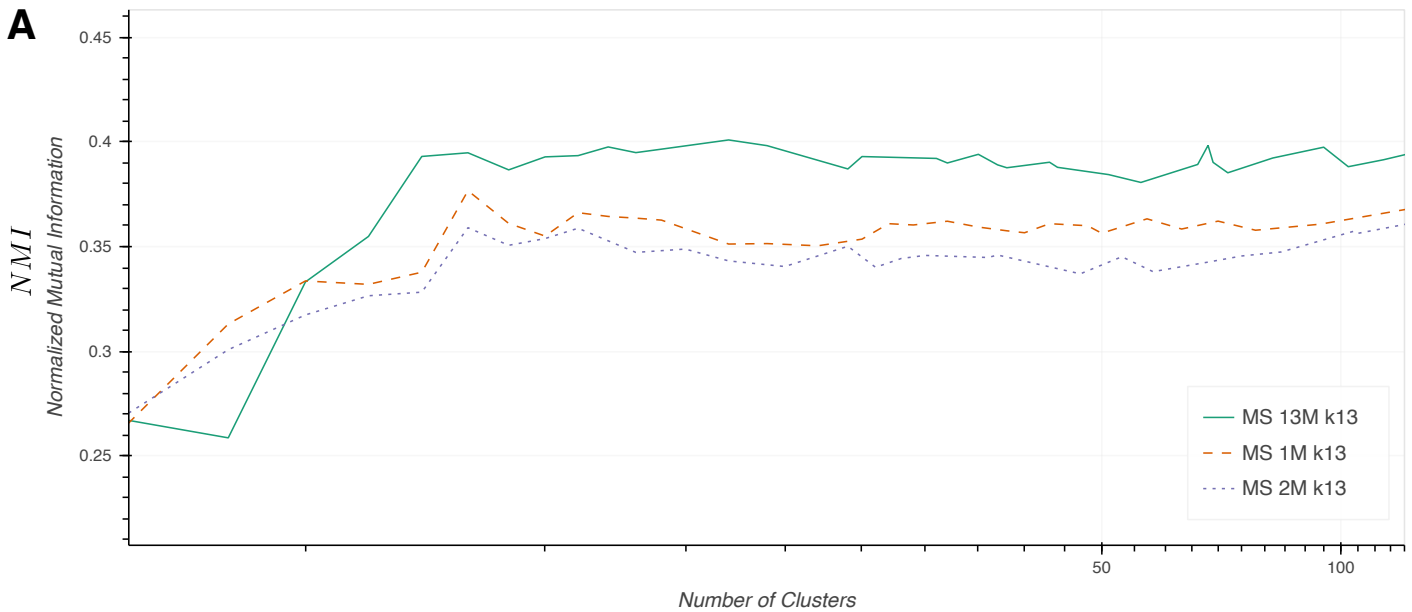**B**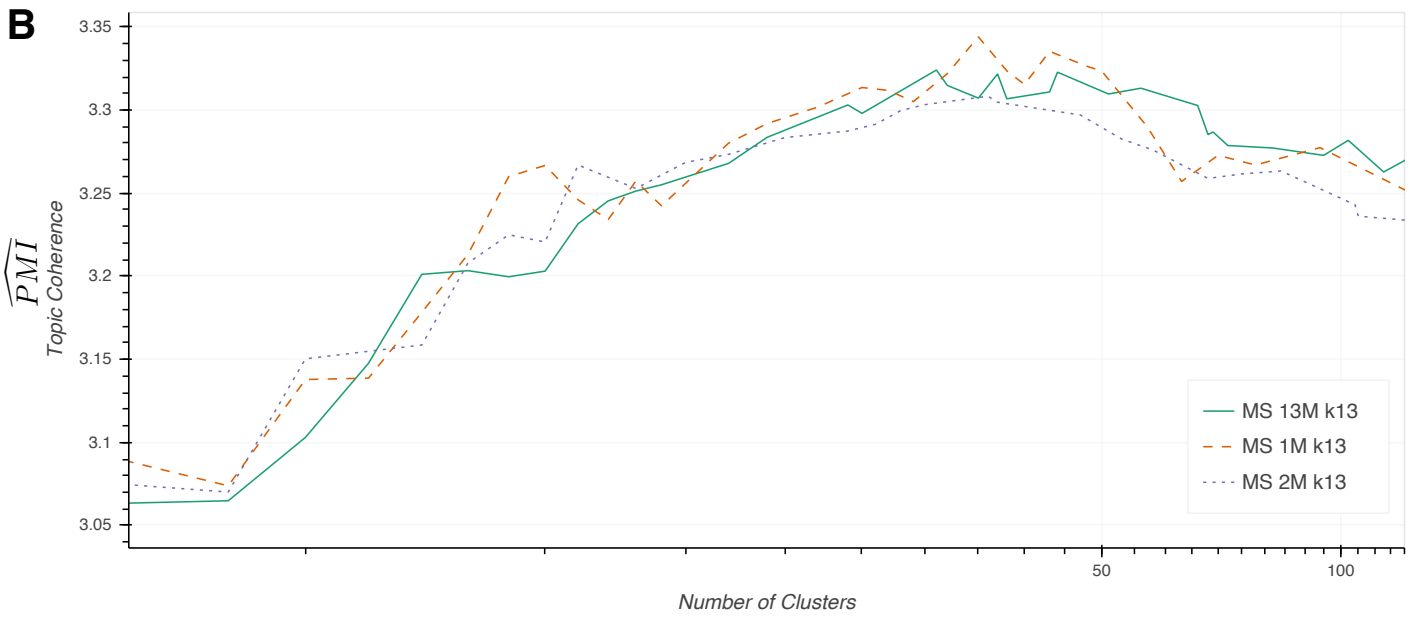

Supplement: Supplementary file 3 — Effect of the corpus size. (PDF 107 kb) [file 41109_2018_109_MOESM3_ESM.pdf]

**A**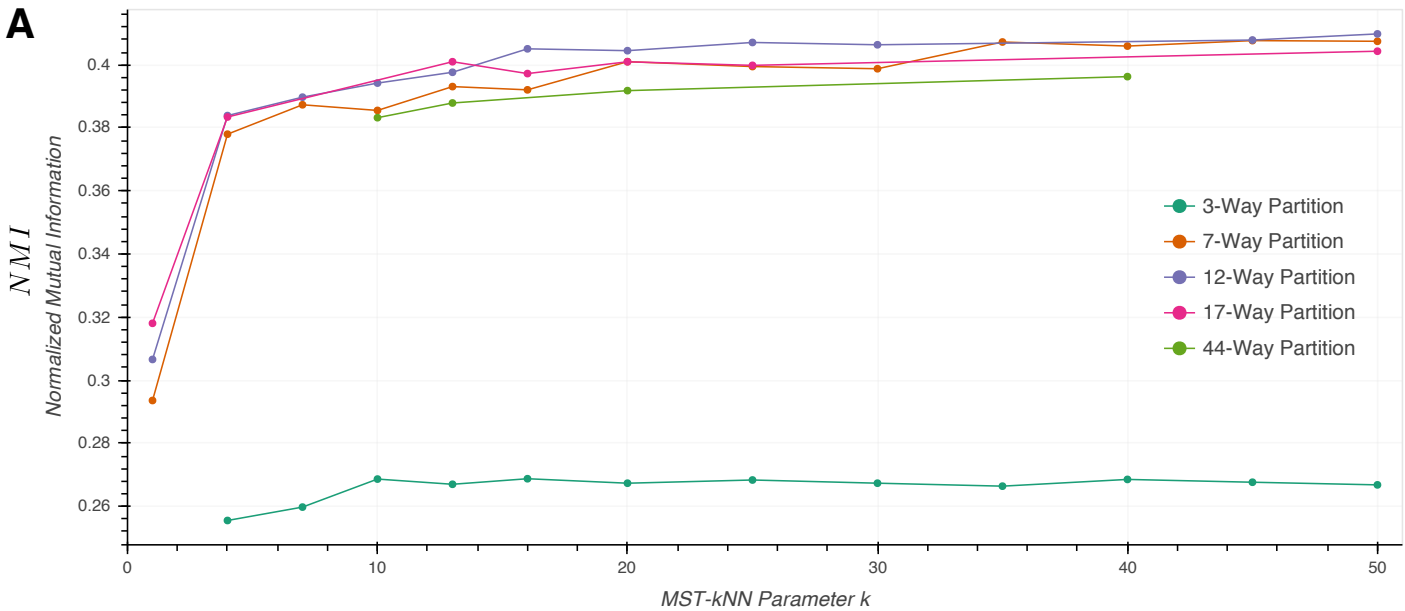**B**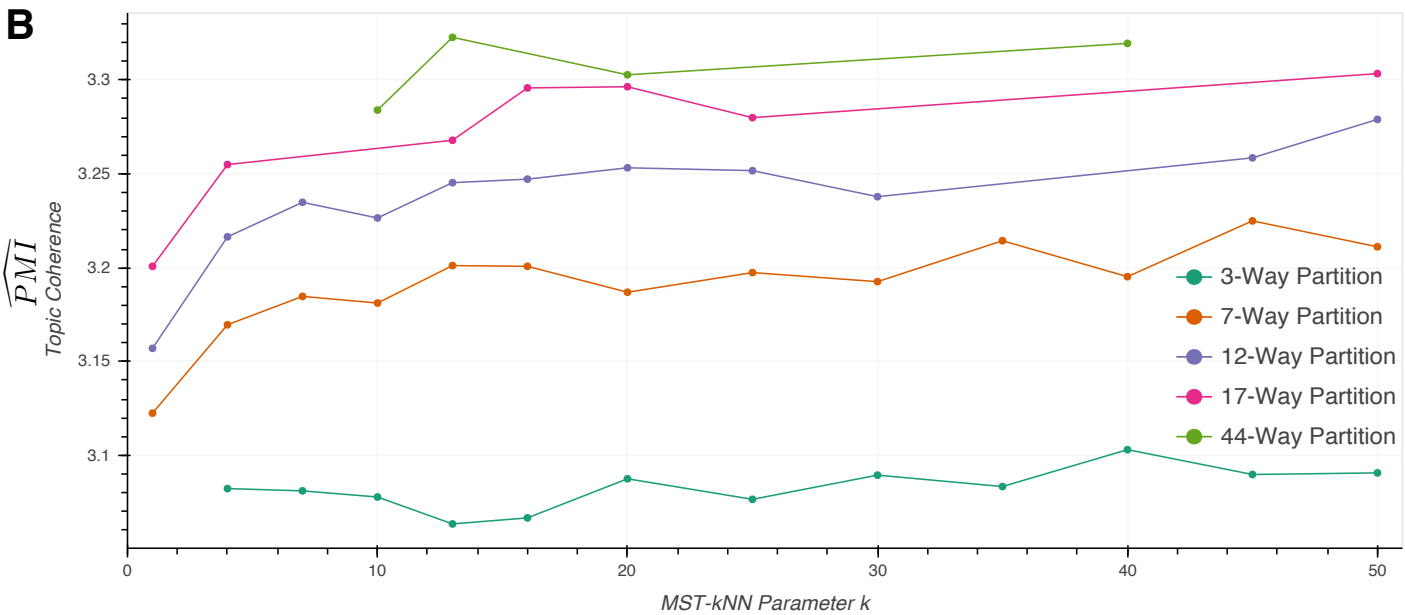

Supplement: Supplementary file 4 — Effect of the sparsification. (PDF 123 kb) [file 41109_2018_109_MOESM4_ESM.pdf]

**A**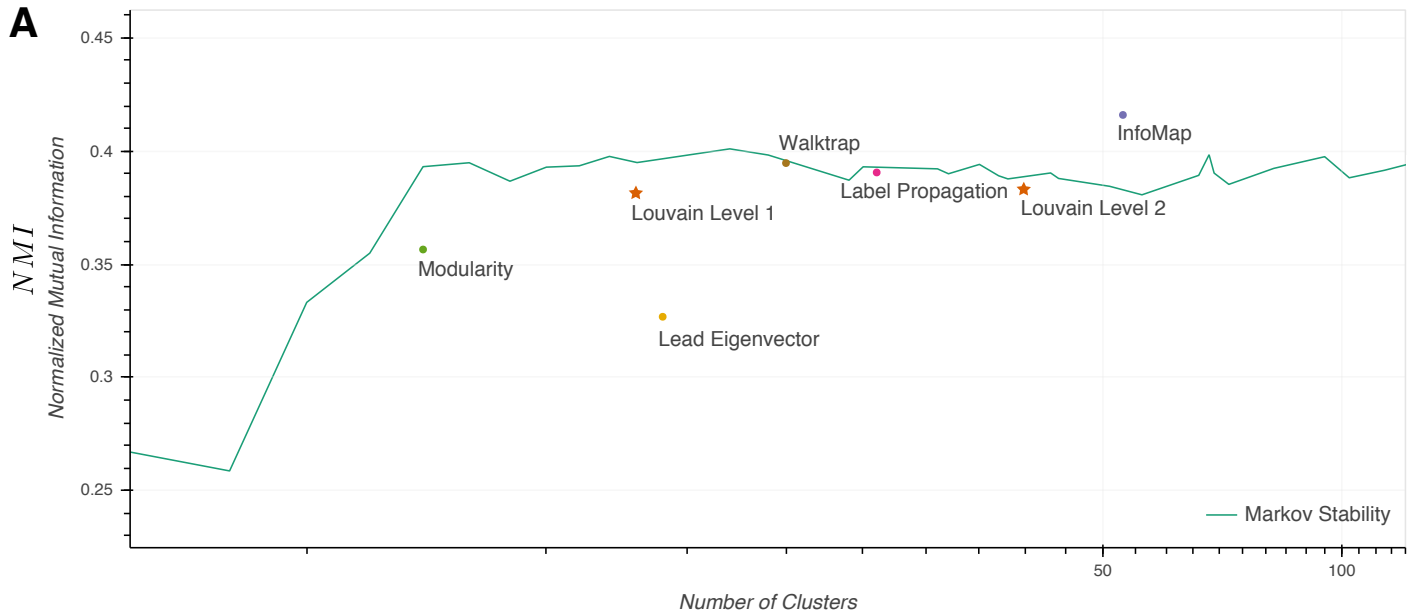**B**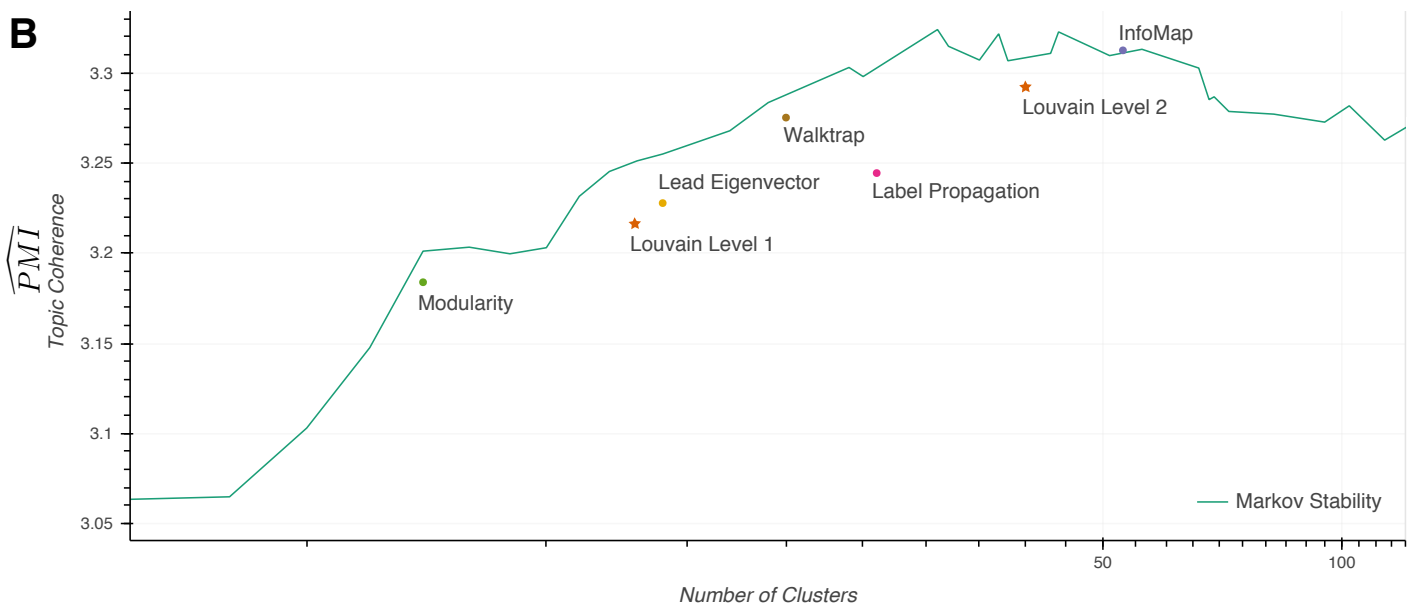

Supplement: Supplementary file 5 — Comparison with other clustering methods. (PDF 103 kb) [file 41109_2018_109_MOESM5_ESM.pdf]
